# Bacterial growth and form under mechanical compression

**DOI:** 10.1038/srep11367

**Published:** 2015-06-18

**Authors:** Fangwei Si, Bo Li, William Margolin, Sean X. Sun

**Affiliations:** 1 Department of Mechanical Engineering and Johns Hopkins Physical Sciences-Oncology Center, The Johns Hopkins University, Baltimore, Maryland 21218, USA; 2 Department of Microbiology and Molecular Genetics, University of Texas Medical School at Houston, Houston, TX 77030, USA

## Abstract

A combination of physical and chemical processes is involved in determining the bacterial cell shape. In standard medium, *Escherichia coli* cells are rod-shaped, and maintain a constant diameter during exponential growth. Here, we demonstrate that by applying compressive forces to growing *E. coli*, cells no longer retain their rod-like shapes but grow and divide with a flat pancake-like geometry. The deformation is reversible: deformed cells can recover back to rod-like shapes in several generations after compressive forces are removed. During compression, the cell elongation rate, proliferation rate, DNA replication rate, and protein synthesis are not significantly altered from those of the normal rod-shaped cells. Quantifying the rate of cell wall growth under compression reveals that the cell wall growth rate depends on the local cell curvature. MreB not only influences the rate of cell wall growth, but also influences how the growth rate scales with cell geometry. The result is consistent with predictions of a mechanochemical model, and suggests an active mechanical role for MreB during cell wall growth. The developed compressive device is also useful for studying a variety of cells in unique geometries.

Bacteria exist in a wide variety of forms, ranging from spheres, rods, and helices to branched, tapered, and flat morphologies^1^,^2^. While the genetic differences that correlate with different bacterial shapes are known, actual molecular mechanisms that connect genes with organismal morphology are less clear. It is thought that a combination of physical and biochemical mechanisms gives rise to the final cell wall shape^1^,^3^,^4^,^5^,^6^,^7^,^8^. In the Gram-negative *E. coli*, a single peptidoglycan (PG) layer in the cell wall is responsible for the rod-like shape. The PG layer is a covalently bonded network of long, rigid glycan strands cross-linked by relatively short and flexible peptide bridges. It is a strong but elastic network that provides mechanical strength to counteract internal turgor pressure and prevent cell lysis^9^,^10^,^11^. With the cooperation of the actin homolog MreB, *E. coli* grows by inserting PG into multiple sites in the lateral cell wall^10^. MreB is essential for the rod-like cell shape since it directly or indirectly recruits and positions PG biosynthesis machinery. It has been shown that when MreB is depleted, cells rapidly stop elongating, increase their diameter and grow with a spherical morphology^12^,^13^,^14^. Existing experiments and biophysical models have demonstrated that MreB also contributes to the stiffness of cell wall and may exert inward forces on the wall, maintaining the rod-like shape and preventing surface wrinkling during cell wall growth^6^,^15^,^16^,^17^,^18^,^19^.

From a mechanochemical prospective, since the PG layer can be considered as a single macromolecule, it has been proposed that the growth dynamics of the cell wall can be understood in terms of a mechanochemical energy^20^. This model predicted that, when nutrient and other variables are held constant, the rate of wall growth is controlled by the change in the cell wall mechanochemical energy. This leads to an explanation of the steady cell radius, which is the stable radius at which the cell wall mechanochemical energy is a minimum. In practice, in standard laboratory culture, rod-like bacterial cells do not change their radius and only elongate. The elongate rate is controlled by many factors, including DNA replication and protein synthesis. Therefore, it is difficult to observe the presence of a steady radius. A different approach is to externally perturb the rod shaped cell and observe how the bacterial cell adapts to perturbations^19^,^21^,^22^,^23^. For example, filamentous *E. coli* cells growing in a curved shape along microchamber walls retain their bent shape when removed from the constraint^21^. Another experiment found that *E. coli* cells can pass through micro channels that are narrower than the cell diameter, and the cell shape became irregular^22^. In both cases, cells recover their rod-like shape after sufficient growth when removed from the confinement. Thus, *E. coli* cell is able to plastically adapt its morphology instead of growing as a straight, cylindrical rod in confined spaces. In addition to geometrical confinement, external mechanical forces have a similar effect on cell shape. It has been shown that when *E. coli* cells are bent by a torque coming from fluid flow, cell grows more on the side under tension, leading to a curved shape that is maintained after the torque is removed^24^,^25^. These experiments show that growth dynamics of the cell can be further examined in these alternative settings.

Here, we carry out microfluidic experiments to quantitatively examine growth rate, division, DNA replication, and protein synthesis in *E. coli* cells under external mechanical compression. We apply long-term, uniform forces on the lateral cell wall and find that the shape of *E. coli* cells reversibly transforms from rod-like to pancake-like. The cell volume and growth rate (volume/time) of cells are nearly insensitive to mechanical compression for reasonable compression depths. Growth of the cell wall (PG synthesis) occurs on the entire cell periphery with no discernible inert poles. The average division time of the pancake-like cells is comparable with normal cells, but the division time shows greater variation. We show that the rate of cell radius of curvature (ROC) change is inversely related to the local ROC. Interestingly, there exists a stable ROC at which the rate of ROC change vanishes. The stable ROC is consistent with predictions of the mechanochemical model. MreB can influence this steady ROC, which suggests a mechanical role for MreB during cell wall growth that influences the final shape of the cell.

## Results

### Design of air-driven microfluidic compression device

**Figure 1:**
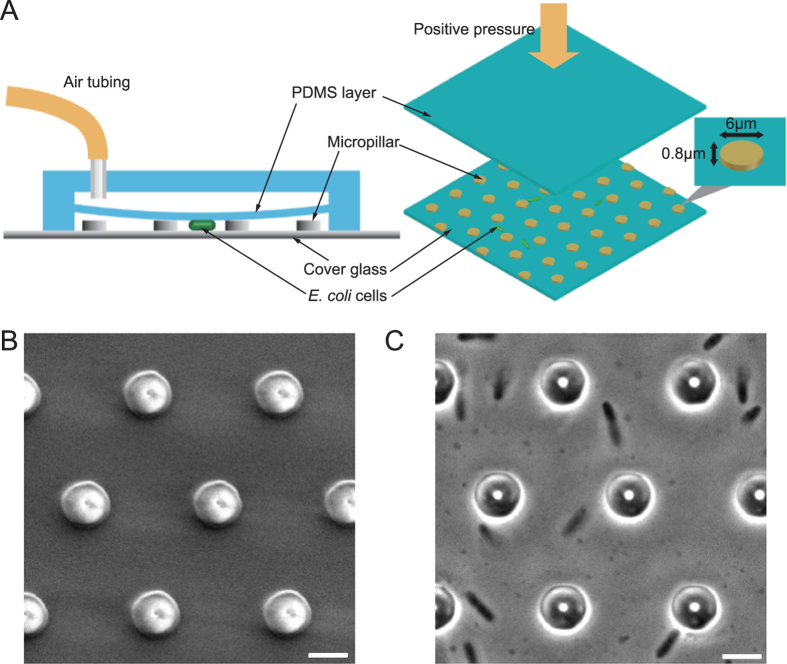
Air-driven microfluidic device applying a compressive force on *E. coli* cells. (**A**) Left: Side view of the device. The device contains two chambers. The upper air chamber and lower cell culture chambers are separated by a PDMS layer of 200 *μ*m in thickness. The PDMS layer is deformed downward to compress cells in the culture chamber when there is positive pressure in the air chamber. Micropillars made by a photoresist are deposited onto the coverglass, which support the PDMS layer when pressure is applied. Right: 3D view of the device. Mircopillars are patterned hexagonally with a distance of 10 *μ*m between pillars. Pillar diameter is 6 *μ*m and typical height is 0.8–0.9 *μ*m. (**B**) Low vacuum scanning electron microscopy image of micropillars on the coverglass. (**C**) Phase contrast image of live *E. coli* cells distributed in the culture chamber between micropillars. Before compression, cells swim and diffuse within the chamber normally. Some cells adhere onto the bottom from the poly-ethylenimine coating. (Scale bars, 5 *μ*m)

Air-driven valve is an easy-to-use method of controlling flows in microchannels, and has been widely used in microfluidic devices and largescale biochips^26^. The deformation of PDMS driven by air pressure can be utilized to apply mechanical forces to cells and tissues^27^,^28^. Here we employed air-driven deformation of PDMS to apply compression forces to bacterial cells. We fabricated a microfluidic device with upper and lower chambers separated by a PDMS layer of 200 *μ*m in thickness. The upper chamber can be inflated by positive air pressure. The lower chamber is where cells were cultured, and was 5 × 5 mm in size and 250 *μ*m in height (Fig. 1A, left). The variable air pressure in the upper chamber deforms the PDMS membrane downward and applies a mechanical force on the *E. coli* cells.

Within this device, the compressive force applied on individual cells can be estimated, but the precise value of the compressive force depends on the pressure in the air chamber, and the thickness and elastic modulus of the PDMS layer. In addition, the elastic modulus of PDMS (2.2 MPa, measured in our experiments) and *E. coli* cells (20 MPa^29^) are of similar order, therefore the assumption that either the PDMS layer or the cell body is rigid is not applicable. Thus, instead of controlling the compressive force, we use a design where we can precisely control the deflection of PDMS by introducing micropillars. Micropillars made by a photoresist were deposited onto the bottom cover glass of the lower chamber, and were used to support the membrane, providing a maximum limit of the PDMS membrane deformation as well as the deformation of the underlying cells. The typical height of micropillar is 0.8–0.9 *μ*m, which is slightly thinner than the average *E. coli* cell thickness (typically 1 *μ*m), and thus achieving a moderate deformation of the cell body. To prevent possible buckling, the diameter of micropillar was set to 6 *μ*m, much larger than its height. All micropillars were patterned hexagonally on the cover glass substrate with 10 *μ*m distance between each pillar. With this approach, PDMS layer sagging can be ignored (Fig. 1A, right).

*E. coli* cells were flowed with LB medium into the culture chamber. To ensure that a number of cells were immobilized during loading and unloading processes of compression, 1% poly-ethylenimine was added with LB medium. A moderate pressure (~5 psi or 34 kPa) in the air chamber was kept constant by a pressure regulator. The downward movement of PDMS layer stopped when the layer contacted micropillars and applied a constant force on the bacterial cells. During compression, a temperature of 37 C° was maintained and fresh LB medium was supplied by a constant flow, thus assuring that *E. coli* cells stay in the growth phase.

### Bacterial cell growth rate, protein synthesis and DNA synthesis are essentially unchanged under compression

**Figure 2:**
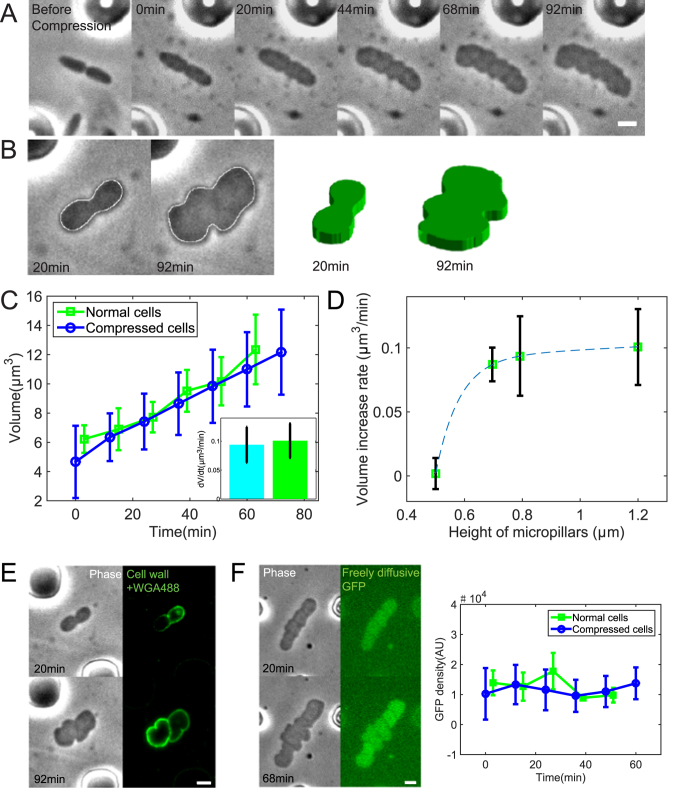
Cell shape and volume changes in *E. coli* cells during mechanical compression. (**A**) Phase contrast images of cell shape evolution before and after compression is applied. (**B**) Outline of cell cross-sectional area under compression and the corresponding 3D view of reconstructed cell shape. (**C**) Volume change of compressed and normal *E. coli* cells within 80 minutes. (n = 7 and 9 for compressed and normal cells, respectively. Error bars indicate standard deviation.) Inset: volume growth rates for compressed and normal cells. The micropillar height is 0.8 *μ*m. (**D**) Volume growth rate as a function of mircopillar height (equal to the thickness of compressed cells). Cells no longer grows when the height is 0.5 *μ*m. (n ≥ 7 for each point. Error bars indicate standard deviation.) (**E**) Cell wall stained by WGA Oregon green 488. (**F**) Left: Phase contrast and fluorescence images of *E. coli* cells expressing freely diffusive GFP under compression. Right: temporal change of GFP density of compressed and normal *E. coli* cells. GFP density was calculated by integrating the total fluorescence intensity of GFP over the cell divided by the cell volume. (n = 5 for both compressed and normal cells. Error bars indicate standard deviation.) (Scale bars, 2 *μ*m)

*E. coli* cells were immobilized when compressed by the PDMS layer (Supplemental movie 1 and 2). The contact region between cells and the bottom cover glass (or the upper PDMS layer) increased immediately after compression, suggesting that cells were mechanically squeezed (Fig. 2A, 1st and 2nd frames). Cells also immediately restored their original shape when we released the pressure after a short (<1 min) compression (data not shown): confirming that the deformation during the initial phase of compression is elastic. When the compression was applied for 60–90 minutes, instead of axial elongation seen in constrain-free cells, compressed cells expanded outward along the whole periphery, including the original pole regions. Cells eventually developed into flatten shapes with ruffled outline and bulges (Fig. 2).

These observations raised two possibilities: that the irregular expansion of the cell wall is due to either cell growth or a physical deformation stemming from a viscoelastic response of the cell wall to mechanical force. To find out which of these mechanisms underlies the long-term response of cell shape change, we first measured the rate of cell volume change for both compressed and normal cells. For the compressed cells, the volume was calculated by multiplying the cell-substrate contact area (by a cell-outlining algorithm) by the height of micropillars (Fig. 2B). For the normal cells, volume was obtained by summing the volume of a cylinder and two hemispherical caps. We found (Fig. 2C) similar cell volume increase versus time for both compressed and normal cells. We also fitted the cell size increase by an exponential function *V*_*t*_ = *V*_0_2^*at*^, where *V*_*t*_ and *V*_0_ are the current and initial cell volumes, respectively. *t* is time and *a* is the growth rate. The measured growth rates for normal and compressed cells were similar (Fig. S1A), suggesting that the expansion rate of compressed cells is comparable to that of normal cells. In addition, we explored cell volume change under different amounts of compression controlled by micropillars of different heights. For micropillars taller than 0.7 *μ*m, the rate of volume increase was close to that of normal cells. However, the rate of volume increase was near zero when micropillar height is 0.5 *μ*m (Fig. 2D). The reason for this is not known, but may be related to altered functions of FtsZ and ribosome under pressure^30^,^31^.

We also examined cell wall synthesis in compressed cells. The cell wall was labeled by fluorescent wheat germ agglutinin (WGA-oregon green 488 conjugate, or WGA488). During compression, fresh LB medium with 10 *μ*g/ml WGA488 was constantly supplied to visualize any newly synthesized cell wall. Fig. 2E shows that the cell periphery expanded with continuous fluorescence without obvious gaps. From these results, we conclude that the observed shape changes are due to alterations in cell wall growth dynamics.

To further check if the compressed cells are maintaining normal physiological processes, we investigated protein and DNA synthesis. *E. coli* cells expressing freely diffusible green fluorescence protein (GFP) from exogenous plasmids were examined. During compression, GFP fluorescence was not disrupted in irregular cells (Fig. 2F, left and supplemental movie 3). The GFP fluorescence density, calculated as the ratio of total fluorescence intensity to the cell volume, was also constant as a function of time for both compressed and normal cells, indicating that the cytoplasmic concentration of GFP is constant in compressed and normal cells (Fig. 2F, right). We also examined an endogenous protein, chromosomally fused MreB-mcherry, which is the only source of MreB in the cell. During compression, the intensity of MreB-mcherry was also constant (Fig. S1B), demonstrating that both exogenous and endogenous proteins are expressed at comparable rates in compressed and normal cells.

Furthermore, we asked whether the DNA replication activity was changed during compression. We labeled the bacterial DNA with Hoechst 33342 (10 *μ*g/ml) to measure the DNA content in growing cells under compression. Fig. S1C shows that the measured DNA content density (the ratio of total DNA content to cell volume) was higher in compressed cells than in normal cells. This might be due to increased permeability of the cell envelope or altered chromosomal structure in compressed cells. Nevertheless, it shows that, in compressed cells, the DNA content increased with increasing cell volume, indicating the DNA replication was progressing. We conclude from these results that during compression, *E. coli* cells can adapt their shapes to cope with external compressive force while maintaining their physiological processes such as growth, protein synthesis, and DNA replication.

### Bacterial cells divide under compression with near normal division rate

**Figure 3:**
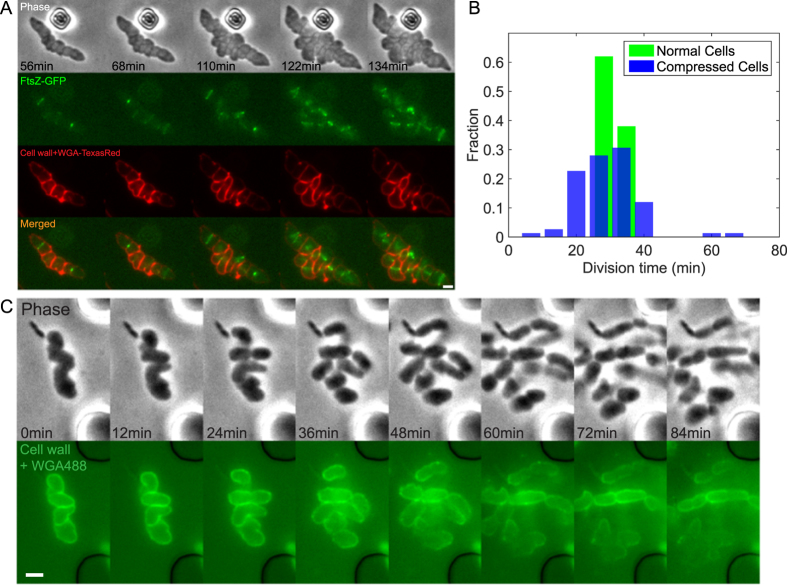
Division of *E. coli* cells under compression. (**A**) *E. coli* cells expressing FtsZ-GFP shows that the FtsZ-ring still forms during cell division. The FtsZ-ring is not always continuous. (**B**) Comparison of division times in compressed and normal cells. The average cell cycle length of compressed cells is not significantly different from the normal cells. However, the standard deviation of cell cycle length of compressed cells is larger. (n = 75 and 71 for compressed and normal cells, respectively) (**C**) Recovery of cell shape after compression is removed. The recovery process occurs together with cell division and elongation. (Scale bars, 2 *μ*m)

In this section, we investigate the process of cell division during mechanical compression. We showed that the cell wall was still being synthesized. Cells formed new septa and were being separated into new cell compartments (Fig. S2). The daughter cells also divided normally. Next we examined the essential protein of cell division, FtsZ, which forms a ring-like structure (FtsZ-ring or Z-ring) at mid-cell during division. In compressed cells, FtsZ-GFP still formed Z-rings, although the ring was typically not continuous around the cell. The Z-ring constricted during cytokinesis and disassembled when daughter cells segregated (Fig. 3A). In normal rod-shaped cells, Z-ring is oriented perpendicular to the axial direction of cell. In compressed cells with irregular shapes without a well-defined long axis, Z-ring still tended to orient perpendicular to the long axis if the cell was slightly elongated. The Z-ring orientation is likely determined by the oscillating minCDE system^32^,^33^, as well as nucleoid occlusion^34^, which both negatively impacts FtsZ assembly.

To identify whether the external force alters the cell cycle time, we measured the division time of compressed cells. In our experiment, one cell cycle is the time between two successive Z-ring disassembly events. The average division time of compressed and normal cells was 29 ± 9 min and 29 ± 6 min, respectively. Therefore the average division time of the compressed cells is comparable to the normal cells. However, the distribution of cell cycle times of the compressed cells was wider than that of normal cells (Fig. 3B).

Recently, the mechanism of generational cell size control was investigated for rod-shaped *E. coli* cells^35^,^36^. Here we can also investigate the added cell size in the compression device. Interestingly, for compression cells, the added cell volume (cell volume at division minus the cell volume at previous birth) was an increasing function of birth volume (Fig. S3A). This is in contrast to the result for cells not under compression, which shows that the added cell volume is roughly a constant. One possible explanation is that the DNA segregation is perturbed under compression. Variations in DNA content as well as DNA density are larger in compressed cells (Fig. S1C), indicating the copy number of DNA could vary substantially. This could also explain the larger variation of cell cycle time (Fig. 3B), although further investigation is needed to fully answer these questions.

### Cell shape recovery after compression is removed

Cell growth dynamics was also monitored when the compression force was removed. The PDMS layer was lifted by removing the pressure in the air chamber, and cells continued to grow and divide. The cell shape gradually transitioned from irregular to rod-like after 2–4 cell generations (Fig. 3C). The cell shape recovery did not occur within a single cell cycle but was accompanied by cell divisions and subsequent cell elongation. This shape recovery is reminiscent of recovery after removal of A22, which depolymerizes MreB. Here we observed that the shape can transition between rod-like to flat pancake-like in a similar manner during application of mechanical forces.

External bending forces have been shown to cause the elastic and plastic deformation of *E. coli* cells^25^. Similar to these previous observations, when compression was removed, the cell shape partially recovered, but largely maintained the pancake-like shape (Fig. S3B). This again indicated that the deformation of compressed cells also consists of both elasticity and plasticity. Moreover, when cell growth was arrested by using Hoechst dye, the pancake-like cells remained deformed (Fig. S1D) and shape recovery no longer occurred. These results are consistent with the idea that cell wall growth is primary driver of cell shape change^5^.

### Sites of cell wall synthesis are co-localized with MreB

To reveal the physical and biological mechanisms underpinning the observed robust cell growth with irregular shape, we studied the dynamics of cell wall and other proteins involved in PG synthesis. Cell wall synthesis can be visualized using pulse labeling of fluorescent WGA^16^. During cell growth, only old parts of the cell wall were stained, but the newly synthesized cell wall did not exhibit fluorescence. Our time-lapsed images of normal cells after pulse labeling showed that fluorescence from the cell poles persisted after several generations (Fig. S4A). This is consistent with the idea that the cell poles remain inert during most of the cell cycle^16^,^29^. Intriguingly, in compressed cells, cell wall growth occurred at midcell and cell poles. Cell wall elements were inserted into existing peptidoglycan network uniformly along the entire cell periphery (Fig. S4B), and MreB was distributed uniformly in compressed cells. Thus, mechanical compression disrupts the organization of the cell poles.

**Figure 4:**
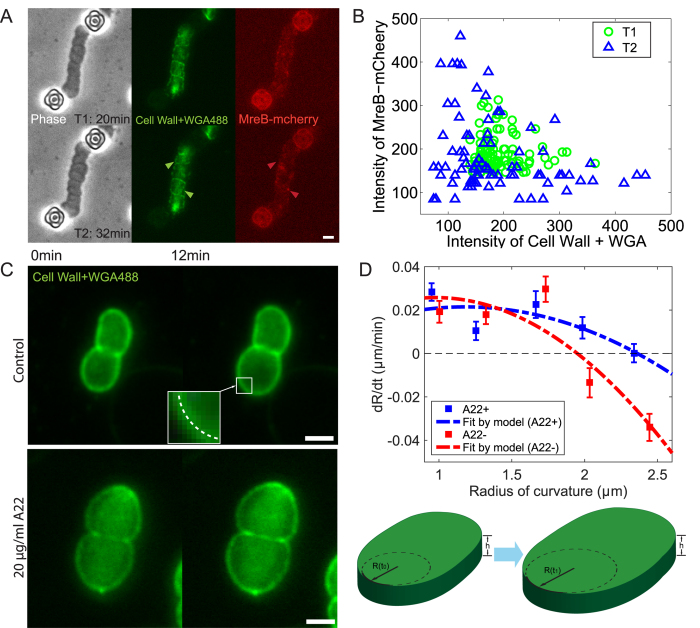
Co-localization of newly inserted cell wall and MreB in compressed cells and the rate of local radius of curvature (ROC) change. (**A**) Pulse-labeled cell wall together with chromosomal MreB-mcherry. Arrows show at later time after compression (32 min), a negative correlation between MreB-mcherry (red) and cell wall+ WGA488 (green) appears (red arrows: MreB assemblies. green arrows: vacancies in cell wall+WGA488). (**B**) The correlation between intensities of MreB-mcherry and cell wall+WGA488 at earlier (T1) and later time (T2) after compression. (n = 6) (**C**) Cells with MreB polymerization inhibited by A22 have different expansion rates under compression. Local radius of cell periphery was measured by fitting a circle (upper, inset) to the cropped arc for both control (upper panel) and cells with 20 *μ*g/ml A22 (lower panel). (**D**) Upper: the rates of local ROC change, d*R*/d*t*, are plotted against local radii for both control (compressed) and cells with 20 *μ*g/ml A22. Experimental data are fitted by model predictions (dashed curves, Eq. 4). Cells in the presence of A22 show a different scaling with respect to *R*. (n ≥ 20 for each point. Error bars indicate standard error of the mean.) The fitted PG Young’s moduli are 22 MPa and 14 MPa for A22− and A22+, respectively. The synthesis rate parameter *γ*, is 3.2 × 10^−6^ and 1.0 × 10^−6^ for A22− and A22+, respectively. The turgor pressures are 140 KPa and 160 KPa for A22− and A22+, respectively. All others parameters are shown in Table S1. Lower: 3D cartoons showing the local ROC changing as cells expand under compression (case of d*R*/d*t* > 0). (Scale bars, 2 *μ*m).

Together with other proteins such as MreC and PBP2, MreB is involved in cell wall synthesis, and has been shown to co-localize with newly inserted cell wall in rod-like cells^37^,^38^. Here, we examined if a similar correlation between MreB dynamics and new cell wall exists in compressed cells. Pulse-labeled cell wall with WGA488 and chromosomal fusion of MreB-mcherry were monitored during compression. After compression was applied, the fluorescence of WGA was initially continuous with spots of MreB-mcherry distributed across the whole cell. Twenty minutes later, fluorescence of WGA became more discrete as cell expanded. In addition, MreB assembly appeared more often at the sites where little WGA488 was observed (Fig. 4A). By plotting the correlation between the normalized intensities of MreB-mcherry and WGA488, we see a clear transition from no correlation at initial time (T1 data) to a negative correlation (T2 data) in Fig. 4B. This indicates that MreB is located at the non-fluorescent sites where new cell wall was synthesized.

MreB has been found to rotate circumferentially in rod-like bacterial cells such as *B. subtilis* and *E. coli*^17^,^39^,^40^. This active rotational motion of MreB is driven by the cell wall assembly and maintains the rod-like shape of the cell^17^. Here we ask if the MreB rotational motion depends on the cell shape. In compressed cells, we found that MreB was moving in a similar manner as in rod-like cells, perpendicular to the cell periphery (Fig. S5A). The velocities and directions of MreB motion had similar distribution as those in rod-like cells (Fig. S5B). Taken together, we found that there is similar degree of coordination between MreB motion and cell wall assembly in compressed cells. Therefore, the cell wall synthesis machinery is not significantly disturbed by external forces.

### Quantitative measure of cell wall growth rates

In addition to cell wall synthesis, MreB also plays a mechanical role in *E. coli* cells^7^,^15^. It has been suggested that MreB can suppress a shape instability in growing rod-like bacteria^7^. To further investigate the mechanical role of MreB, we examined the rate of shape change of *E. coli* cells under compression with normal and partly depolymerized MreB. In particular, we are interested in changes in the radius of cell wall curvature. In normal rod-like cells, the cell radius does not change and elongation of the cell is controlled by metabolic and DNA replication activity in the cell. Under mechanical compression, the cell wall radius of curvature is no longer static but changes with time. The rate of cell wall radius change should also depend on mechanical factors. We investigate this in cells with MreB and in cells treated with A22, which partially disassembles MreB.

For cells with MreB under compression, the shape was identified as the fluorescent periphery labeled with WGA488. The radius of curvature, *R*, was found by fitting a circular arc to a section of cell wall image (Fig. 4C). The rates of *R* change (d*R*/d*t*) were calculated and plotted against the local ROC. Interestingly, for compressed cells with MreB, d*R*/d*t* decreases when *R* increases and reaches negative values around a radius of 2 *μ*m. The positive values of d*R*/d*t* at small radius indicate that the bent cell wall tends to straighten, while the negative values at large *R* suggest a relatively straight cell wall tends to bend as the cell wall grows (Fig. 4D). Furthermore, there exists a fixed point at radius around 2 *μ*m where d*R*/d*t* equals to zero. This fixed point of d*R*/d*t* suggests that a stable ROC exists when cells are subjected to mechanical perturbation.

To quantitatively explain the rate of change of *R*, we employed a biophysical model considered previously^20^. The model describes the relationship between local cell wall growth velocity and the cell wall mechanochemical energy as:


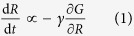


where *R* is the local cell radius specifying the current cell shape, *G* is the cell wall mechanochemical energy^20^ and *γ* is a constant proportional to the cell wall synthesis rate. *γ* does not depend on cell wall geometry, and therefore scales the overall rate of cell wall change. The cell wall energy *G* can be calculated as


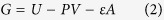


where *U* is the mechanical deformation energy of the cell wall, *PV* is the work done by the current turgor pressure (*P*), *V* is the current cell volume, and *εA* is the chemical free energy change of adding new cell wall (*ε* can be thought of as the chemical bond energy per unit surface area of the cell wall). The compressed cell is flat and pancake shaped. This shape is well described by two flat cell wall layers on top and bottom of the chamber combined with a thin lateral cell wall. Since the energy *G* can be written as a sum (or integral) over different cell wall sections, the local cell wall growth can be described by changes in the local ROC of the flat cell wall layer, *R*. Thus, the relationship between the mechanochemical energy of local cell wall and ROC can be estimated as:


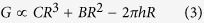


where *C* and *B* are two parameters related to pressure, the mechanical properties and thickness of the PG layer (Supplemental Material (SM)), and *h* is the height of the compressed cell which is equal to the height of the micropillars. Therefore, the rate of change of *R* can be estimated as


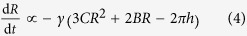


Note that the parameters in the model such as the cell wall Young’s modulus, turgor pressure, chemical energy *ε*, are all potentially controlled by the cell, and may vary in time as the cell is being compressed. The modulus will also depend on whether there are additional mechanical reinforcements from MreB. As shown in the SM, the parameter *C* should depend on cell wall properties, and possibly forces from MreB, and is not known. Parameter *B* depends on the effective turgor pressure and is also not known. However, the scaling relationship with respect to the cell radius of curvature, i.e., d*R*/d*t* proportional to *R*^2^, should remain valid. By fitting unknown parameters to the data (Table S1), we find that within the range of *R* = 1 − 3 *μ*m, d*R*/d*t* decreases when *R* increases, and d*R*/d*t* reaches zeros at *R* ≈ 2 *μ*m. The model predictions are in accord with our experimental results in Fig. 4D.

Treating *E. coli* cells with A22 would partially inhibit MreB polymerization^41^ and reduce any mechanical forces from MreB. In our experiment, a moderate concentration of A22 (20 *μ*g/ml) was added, and cells were then compressed and similarly grew into a pancake-like shape (Supplemental movie 4). The rates of *R* change were also measured, and we also found that d*R*/d*t* decreased as *R* increases (Fig. 4D). However, the quantitative results are different from the cells with intact MreB: d*R*/d*t* reaches zeros at larger values of *R* (Fig. 4D). This indicates that the cell wall prefers to relax to a straighter configuration when MreB is disassembled. We know that in rod-shaped cells, MreB is functioning to maintain the rod-like shape, preventing cell wall from bulging. And for compressed cells with irregular shape observed here, MreB also functions to restrict the overall cell size, preventing cells from over-expansion, in line with our model. According to Eq. 4, when MreB was inhibited, both *C* and *B* are changed. If MreB only affects PG synthesis rates, then the magnitude of d*R*/d*t* would change, but not the scaling with respect to *R*. Therefore, MreB must have a mechanical influence during cell growth, and within the framework of our model, results are consistent with the interpretation that MreB changes mechanical stiffness of the cell wall and/or the cell turgor pressure. Indeed, fitting the model prediction to experimental data clearly shows this (Table S1). The best fit for A22− data corresponds to a PG Young’s modulus of about 22 MPa compared to 14 MPa for A22+ data. The fitted turgor pressure is 140 kPa in A22− cells versus 160 kPa for A22+ cells. The the cell wall synthesis rate for A22− data is about 3 times higher than that of A22+ (Fig. 4D). Therefore, MreB likely serves a mechanical role during cell wall growth. Moreover, it was found recently MreB preferentially binds to regions of negative curvature^16^, which also suggests an active role for MreB.

Note that as compression continues, the cell may actively adapt cell properties associated with variables *C* and *B*. Therefore the stable radius can vary with time. Therefore, to make a consistent comparison between cells with and without A22 addition, the data in Fig. 4 are collected at the same time point after 15 min of compression.

## Discussion

In this paper, we investigate bacterial cell growth dynamics under mechanical compression. We find that *E. coli* cells no longer maintain their rod-like shape when subjected to mechanical compression, and gradually develop into irregular pancake-like shape. On short time scales, the deformation would dramatically alter the distribution of the mechanical stress in cell wall. Our finite element simulation shows that the stress would concentrate at the periphery of flattened cell (Fig. S6). Such stress concentration could create many defects in the cell wall, which could be seen in the cells whose cell wall is not expanding continuously, or cells where membrane blebs have developed (Fig. S7). These defects are present with higher density at the side wall of the compressed cells, creating new bind sites for insertion of new glycan chains^20^. This leads to uniform cell expansion along the whole cell periphery. We also show that the physiological processes of cells are largely undisturbed by the external force, and protein and DNA synthesis are progressing normally in these compressed cells. Moreover, the mechanical compression influences bacterial growth in different ways from changing external hydrostatic pressure^42^. Bacterial cells likely can adapt to a wide range of hydrostatic pressures by adjusting turgor pressure through active pumping of ions^43^. However, under mechanical compression, direct compression of the chromosome and protein macromolecules could completely arrest cell growth (micropillar height <0.5 *μm* in Fig. 2D).

On longer time scales, the sudden stress change in the cell wall is relieved by new cell wall growth. This type of plastic deformation due to growth under external force has been discussed before. The subsequent growth dynamics, according to the mechanochemical model, should depend on the current geometry of the cell. Indeed, we find that under compression, the long term growth dynamics depends on the local curvature of the cell wall, and quantitative results are consistent with scaling results based on a mechanochemical model proposed previously.

In compressed cells, we find that MreB is still functioning normally and catalyzing cell wall growth. MreB is co-localized with newly inserted cell wall in compressed cells, similar to what was found in rod-shaped cell^16^. Therefore, MreB movement and function in cell wall synthesis are robust, regardless of the cell shape. In addition, we find that the presence of MreB alters the scaling of growth dynamics with respect to the local curvature. If MreB’s role is entirely biochemical, then the rate of cell wall growth described by the parameter *γ* in Eq. 1 would change, but the scaling with respect to cell wall geometry should not change. Instead, we find that MreB alters the growth rate as a function of cell wall curvature, implying that MreB affects relative magnitudes of parameters such as *C* and *B* in Eq. 4. These parameters depend on the local cell wall stiffness and internal turgot pressure. Therefore, this result is convincing evidence that MreB not only biochemically catalyzes PG insertion, but also alters mechanical environment of the cell wall.

We also find that there exists an upper limit in cell size when bacterial cells are under compression. Our experiment and theoretical model show that there may exist a stable local radius at which PG synthesis is in a dynamic equilibrium. However, this stable radius depends on local cell wall mechanical properties and any other forces acting on the cell wall. Given that the cell wall may be heterogeneous with spatially varying defects, a fixed stable radius is likely difficult to resolve. Nevertheless, our work, together with other experiments on cell wall growth dynamics under external forces, show that mechanical forces do influence cell wall growth dynamics and geometry of the cell wall in bacteria. MreB alters mechanical forces on the cell wall or mechanical stiffness of the cell wall. In addition, the mechanochemical picture should be valid in any living biomaterial with active growth and turnover. Our experimental approach can be extended to examine other situations as well.

## Methods

### Bacterial strains and growth conditions

The bacterial strain used for measurement of cell volume, radius of curvature, DNA content and cell division was WM2724, a lac- derivative of E. coli MG1655 (WM1074) that expresses ftsZ-gfp from an ectopic site on the chromosome under control of the IPTG-inducible trc promoter. The strain for measuring protein synthesis was WM3497, a derivative of WM1074 that carries plasmid pDSW209, which expresses gfp only. The strain for measuring colocalization of MreB with areas of cell wall synthesis was WM4235, which carries an mreB-mCherry-mreB sandwich fusion at the native mreB locus^44^. WM1283, harboring a thermosensitive plasmid expressing ftsZ and an ftsZ chromosomal null mutation, was shifted to 42 °C to inhibit cell division.

WM2724 and WM3497 were cultured in LB broth overnight at 37 °C, whereas WM4235 was cultured at 30 °C. One hour prior to microscopy, cell cultures were diluted and grown until reaching an OD600 of 0.1. IPTG was then added to WM2724 (0.05 mM) and WM3497 (0.5 mM) to induce FtsZ-GFP or GFP, respectively.

To stain the cell wall, wheat germ agglutinin and oregon green 488 or Texas Red conjugate (WGA488 or WGA-TexasRed, Life Technologies) with final concentration of 10 *μ*g/ml was added with diluted cell culture 10 min prior to microscopy, also together with fresh medium. To stain the DNA, Hoechst 33342 (Life Technologies) with final concentration of 10 *μ*g/ml was added with fresh medium after cells were compressed into irregular shapes.

### Preparation of microfluidic devices

Molds to print the culture chamber and air chamber were fabricated by negative photoresist (SU8-2100, MicroChem Corp.). Typical soft lithography procedure was applied to fabricate our microfluidic devices; 200 *μ*m thick layer of PDMS (1:10 of agent to base, Sylgard 184, Dow Corning Corp.) was spun onto the mold of culture chamber and 7 mm thick PDMS was poured onto the mold of air chamber. Both layers with half-cured PDMS was carefully aligned and then baked until completely cured. Micropillars were fabricated by patterning positive photoresist (s1813, MicroChem Corp.) onto pre-cleaned cover glass (premium cover glasses, Fisher Scientific), and the height of micropillars was measured by profilometer (Dektak IIA) after every experiment. The PDMS and coverglass were bonded after oxygen plasma treatment and baked overnight for use.

Before the experiment, 1% poly-ethylenimine (Sigma-Aldrich, Inc.) with LB medium was added into culture chamber, left standing for 1 hour for coating. Diluted cell culture was then injected into culture chamber through tubing, with some cells immobilized by the poly-ethylenimine coating. Fresh LB medium was then constantly pumped through chamber to assure exponential growth of cells.

### Microscopy and data analysis

All microscopy was done on Nikon TE2000 microscope with phase contrast and epifluoescence. Before compression, cells were allowed to grow at proper temperature in an incubator box (live cell unit, Pathology Devices) for 20 minutes to reach exponential growth. During compression, multiple positions within the contact region in the culture chamber were selected and captured for every 10–12 minutes. After 3 hours of compression, pressure in the air chamber was unloaded, and the capture was continued for another 8–12 hours to record the cell growth when compression was removed. The analysis of cell volume, protein synthesis, DNA content, MreB-cell wall co-localization and change of radius of curvature were all performed by custom algorithms in Matlab (MathWorks, Inc.).

## Additional Information

**How to cite this article**: Si, F. *et al*. Bacterial growth and form under mechanical compression. *Sci. Rep*. **5**, 11367; doi: 10.1038/srep11367 (2015).

## Supplementary Material

Supplementary Information

Supplementary Movie 1

Supplementary Movie 2

Supplementary Movie 3

Supplementary Movie 4
