# Supplementary material for: Bacterial growth and form under mechanical compression
Source: Sci Rep. 2015 Jun 18;5:11367. doi: 10.1038/srep11367 (PMC4471898; doi:10.1038/srep11367)
Supplement: Supplementary Information [file srep11367-s1.pdf]

Supplemental information for "Bacterial growth and form under mechanical compression"

Fangwei Si[1,*], Bo Li[1,*], William Margolin[2] and Sean X. Sun[1,†]

1 Department of Mechanical Engineering and Johns Hopkins Physical Sciences-Oncology Center,

The Johns Hopkins University, Baltimore, Maryland 21218, USA

2 Department of Microbiology and Molecular Genetics,

University of Texas Medical School at Houston, Houston, TX 77030, USA

Mathematical model of bacterial growth

According to our previous work[1], the growth of cell wall could be described by a driving force from the mechanochemical energy change in the cell wall. Therefore, the growth velocity of any geometrical parameter determining cell shape can be described by

$$\frac{\mathrm{d}R}{\mathrm{d}t} \propto -\gamma \frac{\partial G}{\partial R} \tag{S1}$$

where R is a geometrical parameter determining cell shape, G is the cell wall energy[1] and γ is a parameter describing the rate of cell wall synthesis in absence of any mechanical forces. For compressed cells after long-term growth, the cell becomes as two layers of flat cell wall on top and bottom together with a lateral cell wall $0.8 - 0.9\mu m$ in height. Since the cell can only expand horizontally outward, cell growth can be described as the two-dimensional expansion of top and bottom layer of cell wall plus the lateral wall. Locally, the cell wall therefore can be approximated by a cylinder with height $h = 0.8 - 0.9\mu m$ and cross-sectional area proportional to R. We can apply Eq. S1 for the growth of this flat cylinder. For irregular cell shapes, the local curvature R varies. However, local cell wall growth is still well approximated by growth equations for a section of cell wall with local radius R. Thus, we use Eq. S1 to estimate local cell growth rate.

To obtain the total cell wall energy G, we first calculate the strain energy stored in the top and bottom flat layers. Due to symmetry of the flat cylinder, the displacement in the layer in polar coordinates (r, θ) can be written as

$$u_r = u_r(r),\ v_\theta = 0 \tag{S2}$$

The elastic strains are

$$\varepsilon_r = \frac{\mathrm{d}u_r}{\mathrm{d}r},\ \varepsilon_\theta = \frac{u_r}{r},\ \gamma_{r\theta} = 0 \tag{S3}$$

*These authors contributed equally to this work; †Email: ssun@jhu.edu

The stresses are

$$\begin{aligned} \sigma_r &= \tfrac{E}{1-\nu^2}\left(\tfrac{\mathrm{d}u_r}{\mathrm{d}r} + \nu\tfrac{u_r}{r}\right) \\ \sigma_\theta &= \tfrac{E}{1-\nu^2}\left(\tfrac{u_r}{r} + \nu\tfrac{\mathrm{d}u_r}{\mathrm{d}r}\right) \\ \tau_{r\theta} &= 0 \end{aligned} \tag{S4}$$

where E and ν are the Young's modulus and Poisson ratio of the cell wall, respectively. The mechanical equilibrium equation reads as

$$\frac{\mathrm{d}\sigma_r}{\mathrm{d}r} + \frac{\sigma_r - \sigma_\theta}{r} = 0 \tag{S5}$$

Substituting Eq. S4 into S5 leads to

$$\frac{\mathrm{d}^2 u_r}{\mathrm{d}r^2} + \frac{1}{r}\frac{\mathrm{d}u_r}{\mathrm{d}r} - \frac{u_r}{r^2} = 0 \tag{S6}$$

The solution of Eq. S6 is

$$u_r = C_1 r + \frac{C_2}{r} \tag{S7}$$

where $C_2 = 0$ due to the finite displacement at $r = 0$. At $r = R$, we have $\sigma_r = Ph/(2d)$, where h is the height of the micropillars, equal to the height of the flattened cell cylinder. d is the thickness of the cell wall layer. Then we obtain

$$C_1 = \frac{Ph(1-\nu)}{2Ed} \tag{S8}$$

So we have

$$\sigma_r = \sigma_\theta = \frac{Ph}{2d},\ \varepsilon_r = \varepsilon_\theta = \frac{Ph(1-\nu)}{2Ed} \tag{S9}$$

The strain energy in the two flat layers is

$$U_1 = 2\pi \int_0^R (\sigma_r\varepsilon_r + \sigma_\theta\varepsilon_\theta) r\mathrm{d}r = \frac{2\pi P^2 h^2 R^2 (1-\nu)}{2Ed^2} \tag{S10}$$

The stress and strain in the lateral cell wall are

$$\sigma_\theta = \frac{PR}{d},\ \varepsilon_\theta = \frac{PR}{Ed} \tag{S11}$$

The strain energy in the lateral cell wall is

$$U_2 = \frac{\pi P^2 h R^3}{Ed^2} \tag{S12}$$

Therefore, the total strain energy becomes

$$U = U_1 + U_2 = \frac{\pi P^2 h R^3}{Ed^2} + \frac{2\pi P^2 h^2 (1-\nu) R^2}{2Ed^2} \tag{S13}$$

The total energy is

$$G = U - PV - \epsilon A \tag{S14}$$

where $PV = \pi P h R^2$ is the work done by turgor pressure and V is the cell volume and ϵA is the chemical energy. ϵ is the released energy per unit area and $A = 2\pi R^2 + 2\pi h R$ is the surface area of cell wall. So we have

$$G = \frac{\pi P^2 h R^3}{Ed^2} + \left[\frac{2\pi P^2 h^2(1-\nu)}{2Ed^2} - \pi P h - 2\pi\epsilon\right] R^2 - 2\pi h R \tag{S15}$$

Hence, Eq. S1 can be expressed as

$$\begin{aligned}\frac{\mathrm{d}R}{\mathrm{d}t} &\propto -\gamma\left\{\frac{3\pi P^2 hR^2}{Ed^2}+\left[\frac{2\pi P^2 h^2(1-\nu)}{Ed^2}-2\pi Ph-4\pi\epsilon\right]R-2\pi h\right\} \\ &\propto -\gamma(3CR^2+2BR-2\pi h)\end{aligned} \tag{S16}$$

where $C=\frac{\pi P^2 h}{Ed^2}$ and $B=\frac{\pi P^2 h^2(1-\nu)}{Ed^2}-\pi Ph-2\pi\epsilon$. Therefore, C and B have complex dependence on cell wall stiffness, cell height, wall thickness and turgor pressure.

In a growing cell, parameters C and B are expected to vary with space and time, due to complexities of the cell wall structure and possible mechanical contributions from other structures such as MreB. For example, if MreB applies any active forces on the cell wall, then the local Young's modulus would change. However, scaling with respect to geometric parameters such as R should remain the same. Specifically, if we compare growth dynamics with and without MreB, it is possible to resolve mechanical contribution of MreB to cell wall growth. We find that MreB changes relative magnitudes of C and B, therefore indicating that MreB is contributing a mechanical role for cell wall growth.

References

1. Jiang HY, Sun SX (2010) Morphology, growth, and size limit of bacterial cells. Phys Rev Lett 105: 028101.

Supplemental movie 1

E. coli cells are immobilized when compressed by the PDMS layer, and immediately increased the contact area to the bottom cover glass.

Supplemental movie 2

E. coli cells develop into flatten shapes after 60-90 min of compression.

Supplemental movie 3

The fluorescence of freely diffusible green fluorescence protein (GFP) expressed in E. coli cells is not disrupted during compression.

Supplemental movie 4

E. coli cells treated with 20μg/ml A22 also grow into pancake-like shapes and proceed with cell division.

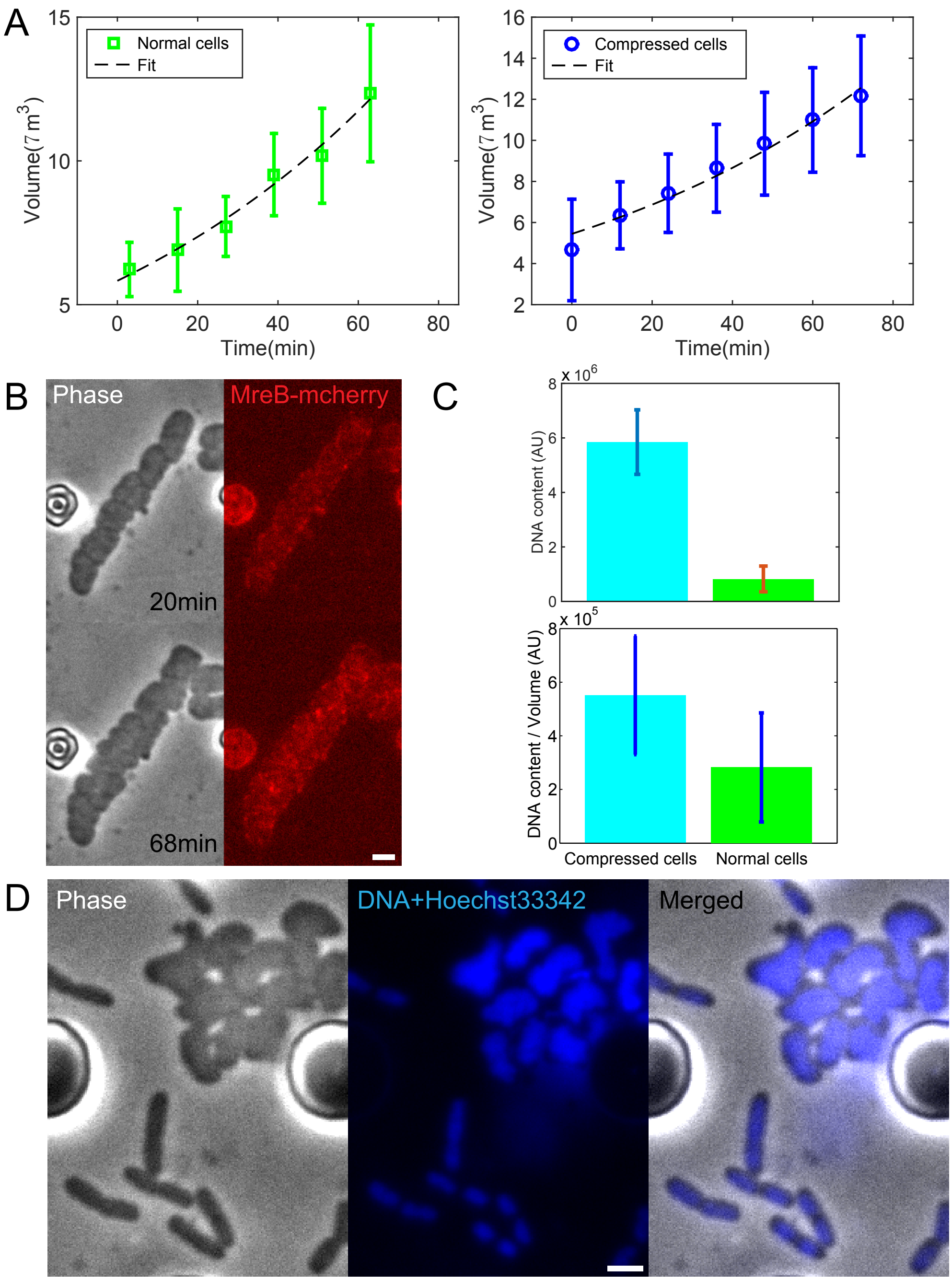

Figure S1: MreB and DNA synthesis in compressed cells. (A) The increase of cell size was fit by exponential function $V_t = V_0 2^{at}$, where $a = 0.0168$ and 0.0167 for normal and compressed cells, respectively (B) Phase contrast and fluorescence images of *E.coli* cells expressing MreB-mcherry (C) *E.coli* cell was stained with Hoechst33342 for DNA, for both compressed and normal cells. (D) Ratios of DNA content to cell volume were compared for compressed and normal cells. DNA content was calculated by integration of fluorescence intensity over whole cell. (n=9 and 10 for compressed and normal cells, respectively. Error bars indicate standard deviation.) (Scale bars, 2μm)

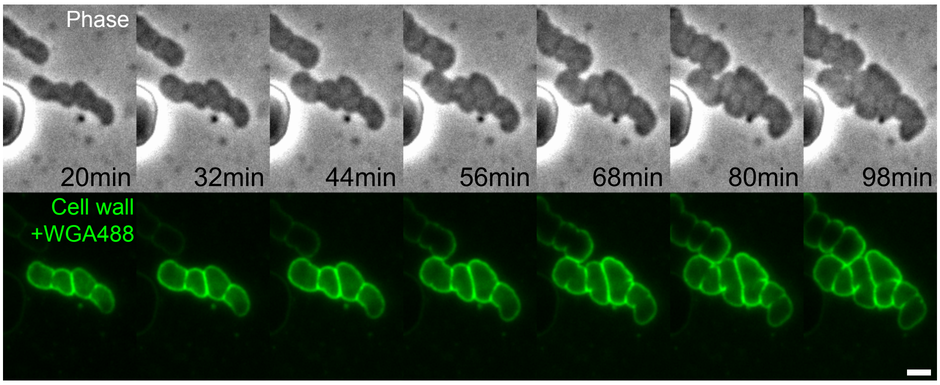

Figure S2: Phase contrast and fluorescence images of $E.coli$ cells stained with WGA488 showing septum formation and cell division occur during compression (Scale bar = 2μm)

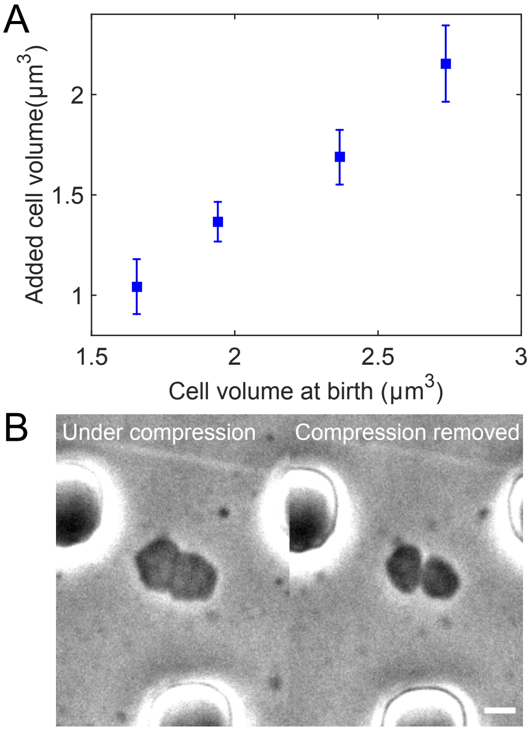

Figure S3: (A) Added cell volume versus cell volume at birth after one cell cycle. (n≥12 for each point. Error bars indicate standard error of the mean.) (B) Cell shape during compression and right after compression removed. (Scale bar = 2μm)

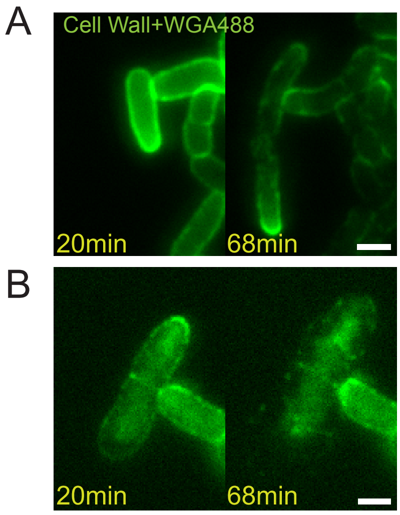

Figure S4: Pulse labeling of cell wall with WGA488. Cell wall keeps continuously labeled at early time and later becomes discrete when new cell wall materials inserted. (A) In normal cells, cellular poles remain labeled when cell is elongating with new cell wall materials inserted at midcell. (B) In compressed cells, cell wall materials are inserted at both polar and midcell sites. (Scale bars, 2μm)

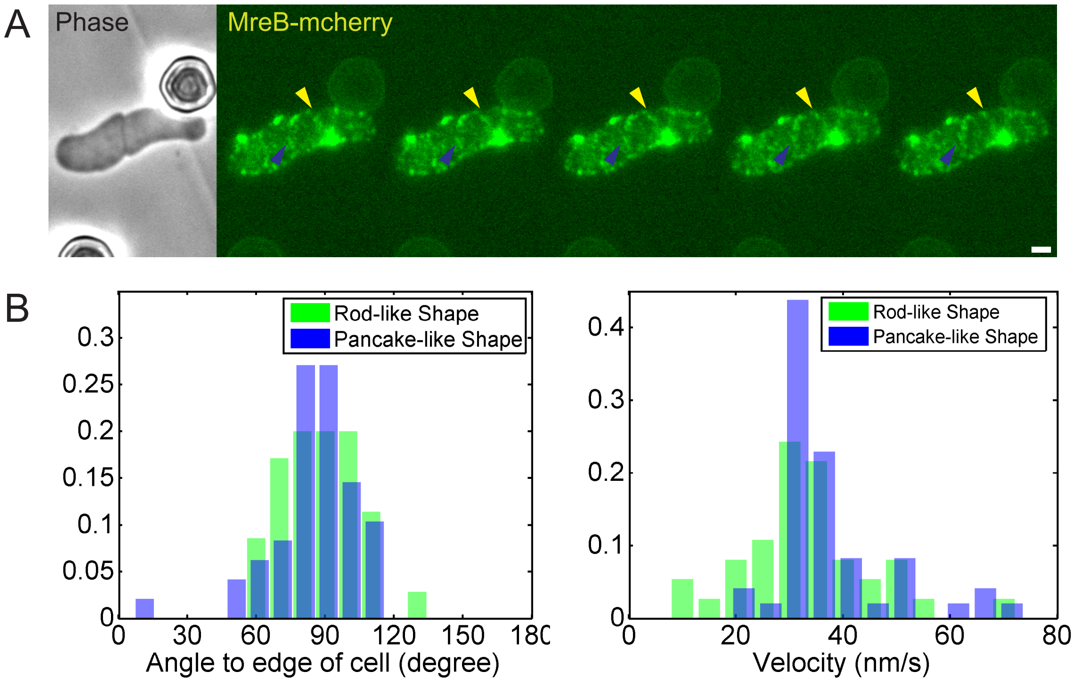

Figure S5: MreB motion in compressed cells (A) Fluorescent spots of MreB-mcherry are moving perpendicular to cell periphery. (Fault color was used for better contrast of images) (B) Probability distribution of angle and velocities of MreB motion. Here the angle was defined by the direction of MreB motion with respect to the nearest cell periphery. (n=48 and 37 for compressed and normal cells, respectively) (Scale bar = 2μm)

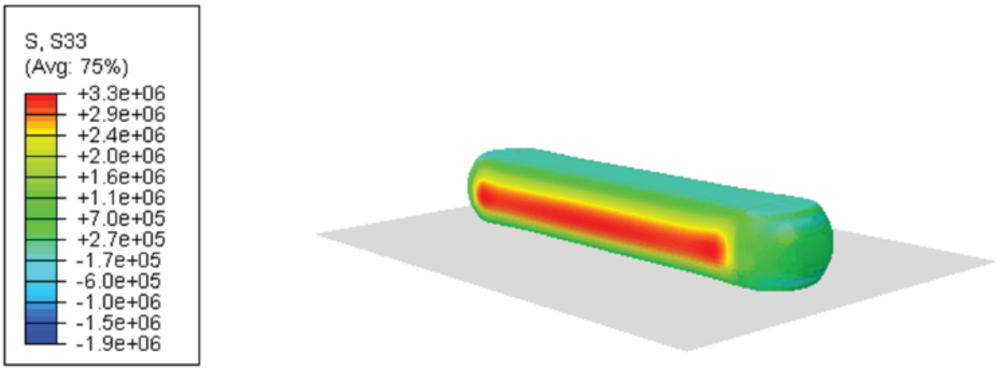

Figure S6: Finite element simulation of an *E.coli* cell compressed by PDMS layer. Tensile stress (Pa) is concentrated at the lateral wall of the flattened cell.

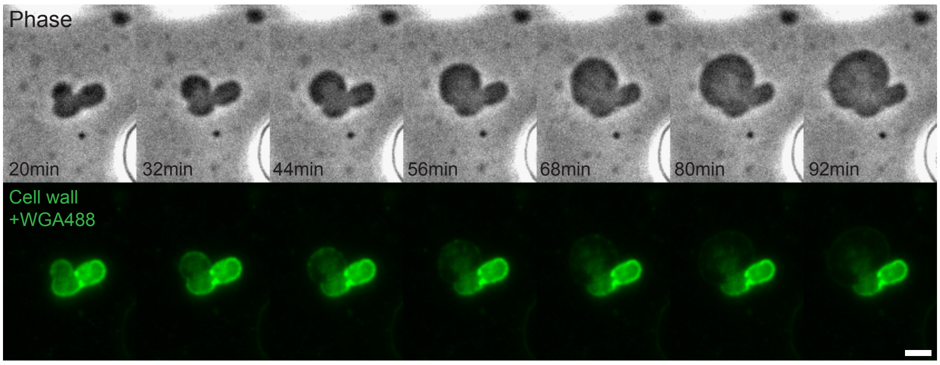

Figure S7: Cells can lyse and develop blebs when compressed. (Scale bar = 2μm)

Fixed parameters	Description	Value for A22-	Value for A22+	Unit
h	Height of the chamber	0.8	0.8	μm
d	Thickness of the cell wall	10^*	10^*	nm
ν	Poisson's ratio of cell wall	0.3^*	0.3^*	
Fitted parameters	Description	Value for A22-	Value for A22+	Unit
ϵ	Chemical energy released per unit area	22	48	$J{\cdot}m^{-2}$
γ	Cell wall synthesis rate	3.2×10^{-6}	1.0×10^{-6}	
E	Young's modulus of cell wall	22	14	MPa
P	Turgor pressure	1.4×10^5	1.6×10^5	Pa

Table S1: Parameters used in the model

*Estimated from reference [1]
